# Development of inexpensive and globally available larval diet for rearing *Anopheles stephensi* (Diptera: Culicidae) mosquitoes

**DOI:** 10.1186/1756-3305-6-90

**Published:** 2013-04-09

**Authors:** Inamullah Khan, Abid Farid, Alam Zeb

**Affiliations:** 1 Nuclear Institute for Food and Agriculture NIFA, G.T Road Tarnab, Peshawar, Pakistan

**Keywords:** Anopheles, Mass rearing, SIT, Wolbachia, Larval diet, Combination diet, Cereals, Legumes

## Abstract

**Background:**

Success of sterile insect technique (SIT) is dependent upon the mass rearing and release of quality insects, the production of which is directly related to the suitability of the diet ingredients used. Commercial diets used for small-scale culture of mosquitoes are expensive and thus not feasible for mass production.

**Methods:**

A series of low cost globally available diet ingredients including, wheat, rice, corn, chickpeas, and beans along with liver, were provided to 4 h larvae (L1) of *Anopheles stephensi* (Liston) to see their effect on fitness parameters including larval duration, percent emergence, survival, adult wing size and female fecundity. Different quantities of the candidate diet ingredients were then mixed together to work out a combination diet with a balanced nutritive value that can be used for efficient rearing of the mosquito larvae at relatively lower costs.

**Results:**

Fastest larval and pupal development and highest survival rates were recorded using a combination diet of bean, corn, wheat, chickpea, rice, and bovine liver at 5 mg/day. The diet is easy to prepare, and much cheaper than the diets reported earlier. The estimated cost of the reported diet is 14.7 US$/ 1.3 kg for rearing one million larvae.

**Conclusions:**

A combination diet with ingredients from cereals and legumes mixed with liver is a low cost balanced larval diet with the potential for use in both small scale laboratory rearing and mass production of *Anopheles* in SIT control programs.

## Background

Malaria is the most deadly insect-transmitted disease. Half of the world's human population lives in malaria affected areas. There are approximately 1 million deaths a year, 250 million cases of clinical malaria each year and about 3.3 billion people at risk of malaria transmission [1]. *Anopheles culicifacies* are primary malaria vectors in rural areas and *An. stephensi* in urban areas of Pakistan [2-6]. Both species breed in clean water habitats, mainly in storage tanks, agricultural drains, irrigation channels, pools, pits puddles and paddy fields [2,6-8]. Application of insecticides is a traditional vector control measure but the use of these chemicals leads to environmental concerns, health hazards and development of resistance in the vector population [9,10]. Therefore, environment friendly approaches for vector control need to be explored. The use of wolbachia for delivering cytoplasmic incompatibility in the field populations and SIT for male sterility are species-specific and environment-friendly control techniques [11-13] that rely on mass-reared males for release and transfer of their sterile, or wolbachia infected sperms to the wild females during mating, causing reduction in the fertility of the females and progressive decline in the target population. To achieve sustainable and affordable production of competitive males for release, mosquito mass rearing is indispensable and an efficient larval diet is a key parameter for the production of healthy male mosquitoes.

Under laboratory conditions, various natural and artificial diets have been tested and favorable food quantity [14] and quality for different mosquito species have been determined. Most common larval diets used in small scale laboratory production include dog biscuits, rabbit feed, bovine liver powder, hog liver, skimmed milk, farex, mouse feed, hay, grass, bread, wheat and cereal infusion, bread crumbs, brewer’s and baker’s yeast [15]. Historically, the major components of mosquito mass rearing diets have been commercial animal diet products because they are easily available in large quantities [16]. For example, *An. albimanis* were mass reared on brewer’s yeast and hog liver [17]. The Koi floating Blend (224 μm-sieved) (Aquaricare, NY) has been used since 2004 at the Insect Pest Control Laboratory (IPCL), Joint Food and Agriculture Organization/ International Atomic Energy Agency for the routine rearing of the *An. arabiensis* colonies. The Koi floating blend (224 um-sieved) is no longer available and a mixture diet with ingredients from bovine liver powder with tuna fish meal has been suggested for production of one million *An. arabiensis* with an approximate cost of US$ 64.30/ 1.4 Kg [18].

Use of locally available low cost diet ingredients for rearing can reduce the cost of rearing *An. stephensi*. We were looking for a combination diet relying more on plant sources because animal derived sources like bovine liver is rich in essential amino acids but lacks vitamins, besides being expensive. A coordinated project was developed with the International Atomic Energy Agency (IAEA) to see the effect of various natural diet ingredients on larval fitness of *Anopheles*. We started with screening individual diet ingredients for their effect on larval survival, developmental duration and adult size (wing length). Then, an additional protein source (yeast) was added to the individual diet ingredients to check for improvement in the diet quality. Finally, different quantities of the diet ingredients were mixed to work out a combination diet with a balanced nutritive value that can be used for efficient rearing of the mosquito larvae at relatively lower costs.

## Methods

### Rearing procedures

A laboratory colony of *An. stephensi* was established at the Nuclear Institute for Food and Agriculture (NIFA), Peshawar during 2007 from larvae that were collected from standing water in irrigation channels of the institute. Larvae were raised in steel trays (38 × 28 × 3 cm) containing 1.0 L of tap water and fed with bovine liver powder. Pupae were collected and placed in small plastic cups and placed inside a new adult cage for emergence. Adults were maintained in transparent Plexiglas cages (40×30×30 cm) with resting places and provided with a 10% (W/V) sugar solution through a feeding apparatus fabricated from 4 cm plastic tubes with mesh at the lower end and a cotton plug soaked in the sugar solution over it. This feeding apparatus was inserted through a hole at the top of each cage. Blood feeding for females were done artificially through mechanically defibrinated bovine blood meal that was offered through a parafilm membrane stretched over an aluminum plate. The temperature of the plate and blood was maintained at 37 ± 2°C through a temperature control electrical device constructed locally at NIFA (Khan unpublished data). Two days after a blood meal, egg cups with blotting paper placed over well saturated wet cotton were put in the cages for oviposition. The rearing conditions of the room were 27 ± 2°C, 60 ± 5% RH; and 12 h: 12 h (light period: dark period).

### Bioassay

For each diet tested, *An. stephensi* eggs were hatched in steel trays (38 × 28 × 3 cm) containing 1.0 L of tap water and a pinch of bovine powder. Thirty, larvae (4–6 hours age) were pipetted and transferred into glass Petri dishes (15 cm diameter) containing 150 ml of tap water. We started with screening individual diet ingredients including corn ‘C’ (*Zea maiz*), mushroom ‘M’ (*Pleurotus ostreatus*), chickpea ‘P’ (*Cicer arietinum* L.), bean ‘B’ (*Phaseolus volgaris)*, rice ‘R’ (*Oryza sativa* L) and wheat ‘W’ (*Triticum, aestivum*), and bovine liver ‘L’ for their effect on mosquito life parameters; larval duration, survival to pupa and to adult stage. In the second series of experiments, baker’s yeast (IMC, Iran) was added in equal quantity to the candidate ingredients (corn, chickpea, rice, bean, wheat, bovine liver) that performed better in the initial screening to see their effect on larval duration, survival, and adult size (wing length). Measurements on the wing length of 10 adults were taken under a stereo microscope from alula to the tip of the wing excluding wing fringes. Finally, different quantities of the candidate diet ingredients were mixed (in equal proportions) to work out a combination diet with a balanced nutritive value that can be used for efficient rearing of the mosquito larvae at relatively lower costs. Life parameters were recorded as mentioned above along with female fecundity.

All the ingredients were dried materials which were ground with a blender (Jack Pot magic JP 739, Japan) and sieved through 100 mesh size (149 microns) test sieve (Wykeham, Farrance Slough England). Five mg of single or single and yeast combined diet was added to the dishes as larval food with one day interval for the first four days and then on a daily basis in the first two series of experiments. In the case of the combination diet, weighed amounts of 2, 3, 4, 5, and 6 mg of each mixture diet were supplied to the larvae with the same routine. Evaporation was reduced by covering dishes with their lids and water was replenished if needed.

### Fecundity

For fecundity tests, about 100 larvae were reared in steel trays (38 × 28 × 3 cm) containing 1.0 L of tap water and each fed with one of the individual diet mixtures. Near to emergence, pupae were put into adult cages (40 × 30 × 30 cm) and the adults were allowed to feed on 10% sugar solution for 6 days. On the 7^th^ day, mechanically defibrinated, bovine blood meal was offered to females through a parafilm membrane stretched over an aluminum plate for blood feeding. Fecundity was determined from five well-fed females that were aspirated from the large cage into a small cage of 24 × 24 × 18 cm size. Two days after a blood meal, egg cups with blotting paper placed over well saturated wet cotton were put in the cages for oviposition. Fecundity was determined by counting the number of eggs laid after the first blood meal. All the experiments were replicated four times.

### Statistical analysis

In the first experiment, developmental parameters were analyzed using ANOVA, using a completely randomized design, followed by Tukey’s Honest Significant Difference (HSD) for mean separation. In the second experiment, yeast when added to a single ingredient was compared for its effects on developmental attributes of *An. stephensi* using a paired t-test. The data for varying quantities of the combination diet (third experiment) were analyzed as Completely Randomized Factorials (using three levels of food and five levels of quantity). In case of a significant food/quantity interaction, the main effects were not analyzed and interaction means were considered. Mean separation was performed using Tukey’s HSD. All the analyses were performed using Statistix 8.1 (Analytical Software, Tallahassee, FL).

## Results

### Single ingredient larval diets

Results from single diet components on developmental attributes of *An. stephensi* are shown in Table 1. Larval duration and time to L1 and adult varied significantly between the different diet (F (7, 24) = 46.1, p < 0.0001). As compared to other ingredients, shorter larval duration was recorded for corn, chickpea and bovine liver. Developmental time was prolonged when the larvae were reared on baker’s yeast and mushroom. Although there was a significant overall variation in survival of L1 to adult (F (7, 24) = 12.5; p < 0.001), the difference was mainly due to the mushroom diet which had a significantly lower survival rate (45.8%).

**Table 1:** **Effect of single diet ingredients on life parameters of *Anopheles stephensi***

**Diet ingredient**	**Larval duration (days)**	**Time L1 to adult (days)**	**Survival of L1 to pupa (%)**	**Survival of L1 to adult (%)**
Bovine Liver	9.75 ± 0.25 cd	11.75 ± 0.25 bcd	83.33 ± 2.00 a	74.17 ± 1.36 ab
Baker’s Yeast *Saccharomyas cerevisiae*	14.25 ± 0.49 a	16.25 ± 0.47 a	69.17 ± 3.19 bc	64.17 ± 1.10 b
Corn *Zea maiz*	8.25 ± 0.25 d	10.25 ± 0.25 d	80.83 ± 1.59 ab	78.33 ± 2.82 a
Mushroom *Pleurotus ostreatus*	15.50 ± 0.64 a	17.50 ± 0.53 a	60.0 ± 0.92 c	45.83 ± 0.2.01 c
Chickpea *Cicer arietinum* L.	9.25 ± 0.24 d	11.25 ± 0.25 cd	79.17 ± 1.44 ab	75.0 ± 1.44 ab
Bean *Phaseolus volgaris*	11.75 ± 0.25 b	13.25 ± 0.25 b	80.83 ± 1.36 ab	76.77 ± 1.44 ab
Rice (*Oryza sativa* L)	11.50 ± 0.29 b	13.50 ± 0.29 b	71.67 ± 2.50 abc	70.83 ± 1.04 ab
Wheat *Triticum aestivum*	11.25 ± 0.25 bc	13.00 ± 0.26 bc	70.83 ± 1.59 bc	69.17 ± 1.35 ab

For each parameter, means followed by the same letter within a column are not significantly different (*P* > 0.05; Tukey’s HSD test using Statistix 8.1); NS, not significant.

### Yeast added larval diets

In two-component larval diets, it was hoped that the addition of yeast would result in better outcomes. Comparison of each ingredient with yeast added is shown in Table 2. Although there was a decrease in developmental time when yeast was added to some of the ingredients, the addition of yeast in most cases did not increase survival to adult stage (t = 0-0.28; p > 0.05). There was decrease in survival to adult stage when yeast was mixed with corn or bean (t = 3.97-5.42, p < 0.001).

**Table 2:** **Comparison of single and yeast added larval diets on life parameters of *Anopheles stephensi *using paired t-test**

**Food ingredient**	**Larval duration (days)**	**Time L1 to adult (days)**	**Survival of L1 to pupa%**	**Survival of L1 to adult (%)**	**Adult size (mm)**	
					M*	F**
Liver + Yeast	10.75	12.75	81.67	75.0	2.96	3.06
Bovine liver	9.75 (t = 1.48, p = 0.19)	11.75 (t = 1.48, p = 0.19 )	83.33 (t = 1.0, p = 0.35 )	74.17 (t = 0.28, p = 0.79)		
Corn + Yeast	10.0	11.90	76.67	69.17	3.02	3,05
Corn	8.25 (t = 3.66, p = 0.01 )	10.25 (t **=** 3.55, p = 0.01)	80.83 (t = 1.99, p = 0.09 )	78 .33 (t = 3.97, p = 0.007)		
Bean + Yeast	9.25	11.25	66.67	65.0	3.02	3.05
Bean	11.75 (t = 4.63, p < 0.001)	13.25 (t = 3.70, p = 0.01)	80.83 (t = 6.76, p < 0.001)	76.77 (t = 5.42 , p < 0.001)		
Chickpea + Yeast	8.25	10.23	77.40	75.83	3.09	3.05
Chickpea	9.25 (t = 2.83, p = 0.03)	11.25 (t = 2.75, p = 02 )	79.17 (t = 0.34, p = 0.74 )	75.0 (t = 0.18, p = 0.86 )		
Rice + Yeast	9.25	11.23	72.50	70.83	3.08	3.05
Rice	11.50 (t = 4.02, p = 0.006)	13.50 (t = 4.04, p = 0.006)	71.67 (t = 0.19, p = 0.86)	70.83 (t = 0.0, p = 1.0)		
Wheat + Yeast	8.50	10.50	77.40	69.17	3.04	3.08
Wheat	11.25 (t = 7.20, p < 0.001)	13.0 (t = 5.0, p = 0.002)	70.83 (t = 2.53, p = 0.04)	67.17 (t = 0.0, p = 1.0)		

*male, ** female.

### Combination larval diets

Results from the quantification of mixed diets are shown in Table 3. There was a significant food/quantity interaction for all the studied parameters. For 2 mg quantity, no difference in all developmental parameters (larval duration, survival, fecundity and male size) was observed. Prominent reductions in larval development from first instar (L1 stage) to pupa and adult were observed with the increase in diet fed from 2 to 5 mg per day. This trend of shorter larval duration was seen in all three diets. Most of the values for all the diets flattened after 5 mg food indicating an excess of food. The Survival rate of larvae to pupal and adult stage (Table 3) also increased with an increase in the food quantity. No significant variation in male size and the number of eggs laid by females was noticed from any diet.

**Table 3:** **Effect of combination larval diets on life parameters of *Anopheles stephensi***

		**Quantity of food (mg) provided/ day**				
**Variable**	**Food (code)**	**2**	**3**	**4**	**5**	**6**
Larval duration (days)	Bean + corn + chickpea + liver (BCPL)	11.0 ± 0.4 a	10.7 ± 0.5 a	8.5 ± 0.3 b	8.0 ± 0 bc	7.7 ± 0.2 bc
	Bean + corn + wheat + chickpea + liver (BCWPL)	10.7 ± 0.5 a	8.5 ± 0.3 b	8.2 ± 0.2 bc	7.0 ± 0 c	7.0 ± 0 c
	Bean + corn + wheat + chickpea + rice + liver (BCWPRL)	10.7 ± 0.5 a	8.2 ± bc	7.0 ± 0 c	7.0 ± 0 c	7.2 ± bc
Time L1 to adult (days)	Bean + corn + chickpea + liver (BCPL)	12.5 ± 0.3 a	12.5 ± 0.3 a	10.5 ±0.3 b	10.0 ± 0.2bc	9.7 ± 0.2 bc
	Bean + corn + wheat + chickpea + liver (BCWPL)	12.5 ± 0.3 a	10.5 ± 0.3 b	10.2 ± 0.2 bc	9.0 ±0.3 bc	9.5 ± 0.3 bc
	Bean + corn + wheat + chickpea + rice + liver (BCWPRL)	12.5 ± 0.3 a	10.2 ± 0.2bc	9.5 ± 0.3 bc	9.5 ± 0 c	9.25 ± 0.2bc
Survival of L1 to pupa (%)	Bean + corn + chickpea + liver (BCPL)	75.8 ± 1.6 c	76.6 ± 1.4 c	78.3 ± 2.1 c	88.3 ± 2.1 a	79.1 ± 1.6 bc
	Bean + corn + wheat + chickpea + liver (BCWPL)	76.6 ± 1.4 c	78.3 ± 2.1 c	79.1 ± 2.1 bc	87.5 ± 1.6 a	87.5 ± 1.6 ab
	Bean + corn + wheat + chickpea + rice + liver (BCWPRL)	76.6 ± 1.4 c	79.1 ± 2.1 bc	89.1 ± 1.6 a	89.1 ± 1.6 ab	88.3 ± 1.4 a
Survival of L1 to adult (%)	Bean + corn + chickpea + liver (BCPL)	73.3 ± 2.3 c	74.1 ± 2.1 c	75.0 ± 2.1 c	87.4 ± 2.5 a	75.0 ± 2.1 c
	Bean + corn + wheat + chickpea + liver (BCWPL)	74.1 ± 2.1 c	75.0 ± 2.1 c	75.8 ± 1.6 bc	86.3 ± 2.1 a	86.6 ± 1.4 a
	Bean + corn + wheat + chickpea + rice + liver (BCWPRL)	74.1 ± 2.1 c	75.8 ± 1.6 bc	88.3 ± 2.1 a	88.1 ± 1.4 a	85.8 ± 1.6 ab
Fecundity (Eggs/female)	Bean + corn + chickpea + liver (BCPL)	101.8 ± 1.5ab	111.3 ± 8.9 ab	115.3 ± 10.5 ab	134.9 ± 3.9 a	117.5 ± 8.7 ab
	Bean + corn + wheat + chickpea + liver (BCWPL)	114.6 ± 6.9 ab	103.9 ± 5.3 ab	104.1 ± 5.1 ab	108.0 ± 6.7 ab	129.7 ± 0.2 ab
	Bean + corn + wheat + chickpea + rice + liver (BCWPRL)	92.5 ± 6.3 b	100.7 ± 1. 8 ab	113.8 ± 9.4 ab	125.4 ± 12.7 ab	105.7 ± 14.7 ab
Male Size (mm)	Bean + corn + wheat + chickpea (BCWP)	2.98 ± 0.13 bcd	2.80 ± 0.11d	3.28 ± 0.14 abc	3.26 ± 0.18 abc	3.32 ± 0.2 abc
	Bean + corn + wheat + chickpea + liver (BCWPL)	2.94 ± 0.15 cd	3.10 ± 0.15 abcd	3.50 ± 0.12 a	3.26 ± 0.18 abc	3.38 ± 0.10ab
	Bean + corn + wheat + chickpea + rice + liver (BCWPRL)	3.14 ± 0.08 abcd	3.26 ± 0.05 abc	3.42 ± 0.20 a	3.50 ± 0.17 a	3.42 ± 0.20 a

Within each variable, means followed by the same letter are not significantly different (*P* > 0.05; Tukey’s HSD test using Statistix 8.1).

Looking at the quality of the diet, comparable results were obtained when 5 mg of 4, 5 or 6 ingredient diets were used, but the cost analysis (Table 4) shows that there is a decline in the cost involved with the addition of each plant derived ingredient. Thus the cost of diet drops from 21.7 US$/ 1.3 kg (with three plant ingredients plus liver) to 14.7 US$/ 1.3 kg (with five plant ingredients plus liver) for rearing one million larvae.

**Table 4:** **Cost analysis of various diet ingredients and their mixtures for rearing *Anopheles stephensi***

**Diet ingredient (Code)**	**Price/ Kg (US$)**	Total cost to rear one million larvae (US$)*	**Availability**
Bovine Liver Powder (L)	63.0	84.0	MP biomedical, Solon, OH
Wheat powder (W)	0.20	0.30	Grocery Shops
Rice Powder (R)	0.8	1.0	Grocery Shops
Corn Powder (C)	0.7	0.9	Grocery Shops
Chickpea powder (P)	0.8	1.0	Grocery Shops
Bean powder (B)	0.6	0.8	Grocery Shops
Bean + corn + chickpea + liver (BCPL)	16.25	21.7	---
Bean + corn + wheat + chickpea + liver (BCWPL)	13.0	17.4	---
Bean + corn + wheat + chickpea + rice + liver (BCWPRL)	11.0	14.7	---

* Cost has been calculated using an amount of 1.3 kg needed to rear one million larvae (an average of 40 mg diet needed to rear a batch of 30 larvae in an average of 8 days).

## Discussion

A series of larval diet experiments were initiated with simple low cost ingredients consisting of various cereals and legumes. From experiments on single diet components, shorter larval periods were recorded for corn, chickpea and bovine liver while larvae feeding on the mushroom diet had the longest larval period. Survival of larvae to the pupal and adult stage was lowest (60 and 45% respectively) for larvae feeding on mushroom in the series of diet ingredients tested.

Nutritional requirements of mosquito larvae are known to include at least 14 amino acids, sugars, polyunsaturated fatty acids (PUFAs) especially C18, C20, and C22, [19-24], sterols, and nucleotides for the larval development, survival and adult flight [25,26], and a minimal concentration of essential vitamins to allow their optimal growth [25,27]. Studies on the vitamin supplements in *Culex pipiens* have shown their significant role in normal growth and survival*.* In related experiments the absence of riboflavin, folic acid, biotin or choline, resulted in few larvae developing to pupae, and most of them died in the 3rd or 4th instar [24,28].

Cereals and legumes can meet several of the dietary requirements of the mosquito larvae. Corn consists of 1-2% lipid with 72-85% unsaturated fatty acids, primarily, oleic acid and linoleic acid. Corn is also rich in vitamin A and has a high protein content [29]. The chickpeas are rich in poly-unsaturated fatty acids, proteins, carbohydrates, B-group vitamins, and certain minerals [30-34]. Both cereal grains and legumes contain varying amounts of carbohydrate, fat, protein, water and minerals [33,35,36]. Thus corn and chickpea alone could support larval development of *An. stephensi.*

In the second experiment, yeast was added to the individual diet ingredients to check for an improvement in the diet quality. The addition of baker's yeast did not lead to any improvement in the measured parameters. It might have been due to scum formation observed in yeast added diets that caused larval mortality and delayed pupal emergence. Based on these observation yeast was not tested in further experiments.

For further improvement in the diet we prepared mixed diets including the components that performed better in the single diet experiment. Different quantities of the mixtures were administered to see the optimum level of each diet mixture. All the three diet mixtures performed well when used in appropriate quantities. Mixing the plant ingredients compensates for nutritional deficiencies present in individual diet components. For example, cereals are reported to have low quantities of the amino acids tryptophan and methionine [37], which are present in legumes such as chick-pea and beans. There was an improvement in larval parameters when the amount of diet administered was increased to more than 4 mg (provided on alternate days for the first four days and then daily). Further increases in the amount fed did not result in any significant improvement. We did not find a significant difference in the fecundity for different quantities of the mixture diets. The difference might have been due to the variation resulting from the protocols used. We estimated fecundity from the output of five females due to handling ease but there is a chance of missing individual difference if some of the females did not oviposit at all.

The wing size of male mosquitoes also did not differ for the mixture diets used in the study. However overall wing size was greater as compared with adults reared on two component diets (Table 2). In a related experiment on mixture diets administered to *An. arabiensis* larvae, the size of L4 larva and male size (wing-length) was reported to be the highest for a mixed diet prepared from natural ingredients [38]. These observations support the hypothesis that single larval diets might be adequate for the completion of larval stages to emergence but may lack appropriate quantities of vitamins and minerals that would positively influence adult size. Other studies confirm the direct effect of parental rearing on the developing embryo [39] or the influence of dietary restrictions on larval and adult development [40]. Although, the objective of the present study was only to look at the effect of different diets on life parameters of mosquitoes and not the effect of larval rearing on offspring, we observed useful clues on the indirect effect of larval diets on adult size. Therefore, further studies should be performed on the effect of diet quality provided to early stages of mosquitoes and their effect on adult life parameters.

We prepared mixed diets by combining bovine liver with ingredients from plant sources (cereal and legumes) that are rich in essential amino acids, saturated and unsaturated fatty acids, minerals and vitamins [37,41]. The inclusion of bovine liver, an animal-derived protein, also ensures the presence of at least 14 essential amino acids, asparagine, arginine, glycine, histidine, isoleucine, leucine, lysine, methionine, phenylalanine, proline, serine, threonine, tryptophan, and valine [18]. The reason that our mixed diets performed better might be due to the high/ balanced nutritive values of the ingredients present in combination diets. Further experiments on the standardization of this diet for mass rearing of several *Anopheles* and *Aedes* species need to be carried out prior to its use on a large scale.

Taking into account the life parameters, comparable results were obtained when 5 mg of 4, 5 or 6 ingredient diets were used. Looking at the cost analysis (Table 4) there is a decline in the cost involved with the addition of each plant derived ingredient thus the cost of diet drops from 21.7 US$/ 1.3 kg (with three plant ingredients plus liver) to 14.7 US$/ 1.3 kg (with five plant ingredients plus liver) for rearing one million larvae. This is because the relative cost of plant derived ingredients is much lower than that of bovine liver and addition of more plant derived ingredients reduces the amount of bovine liver used per kg of the final diet mixture. Therefore we recommend the six component diet (BCWPRL) as the most cost effective balanced diet for rearing the larvae of *An. stephensi*.

Results from related studies on diet development using commercial ingredients for *An. arabiensis*[18] have demonstrated the usefulness of mixture diets from bovine liver, tuna meal and vitamin mix with an estimated cost of US$ 64.30/ 1.4 kg of diet for production of one million mosquitoes [42]. The diet reported here can be used to rear one million mosquito larvae of *An. stephensi* at an average cost of US$ 15 only. The prices calculated here are based on small quantity orders and would probably be reduced significantly when ordering the bulk quantities needed in a mass-rearing facility.

## Conclusions

The duration of the larval period from single component diets improved from 8.25 days for corn in single diet to 7.0 days in a mixture of bean, corn, wheat, chickpea, rice, and liver (BCWPRL). Survival rate from L1 to adult stage also improved from the highest (78%) in single diet experiments to 88% survival when fed 4 mg of the BCWPRL combination diet. Further increase in diet quantity from 4 to 6 mg per day did not improve biological parameters. Regarding price, the six component diet reported here is economical and has the potential to be tested for rearing other mosquitoes. Based on these findings, we recommend the use of natural diet ingredients in larval diets for successful production of quality insects in an SIT program.

## Competing interests

The authors declare that they have no competing interests.

## Authors’ contributions

The initial experimental set up was developed, executed and draft of the manuscript was prepared by I. Khan. Farid, A. performed statistical analysis and helped in the development of the manuscript. Alam Zeb performed the final review of the manuscript. All authors approved the final version of the manuscript.
